# Insights into the Molecular Evolution of HslU ATPase through Biochemical and Mutational Analyses

**DOI:** 10.1371/journal.pone.0103027

**Published:** 2014-07-22

**Authors:** Kwang Hoon Sung, Hyun Kyu Song

**Affiliations:** Department of Life Sciences, Korea University, Seoul, Korea; Wuhan University, China

## Abstract

The ATP-dependent HslVU complexes are found in all three biological kingdoms. A single HslV protease exists in each species of prokaryotes, archaea, and eukaryotes, but two HslUs (HslU1 and HslU2) are present in the mitochondria of eukaryotes. Previously, a tyrosine residue at the C-terminal tail of HslU2 has been identified as a key determinant of HslV activation in *Trypanosoma brucei* and a phenylalanine at the equivalent position to *E. coli* HslU is found in *T. brucei* HslU1. Unexpectedly, we found that an F441Y mutation in HslU enhanced the peptidase and caseinolytic activity of HslV in *E. coli* but it showed partially reduced ATPase and SulA degradation activity. Previously, only the C-terminal tail of HslU has been the focus of HslV activation studies. However, the Pro315 residue interacting with Phe441 in free HslU has also been found to be critical for HslV activation. Hence, our current biochemical analyses explore the importance of the loop region just before Pro315 for HslVU complex functionality. The proline and phenylalanine pair in prokaryotic HslU was replaced with the threonine and tyrosine pair from the functional eukaryotic HslU2. Sequence comparisons between multiple HslUs from three different biological kingdoms in combination with biochemical analysis of *E. coli* mutants have uncovered important new insights into the molecular evolutionary pathway of HslU.

## Introduction

HslVU is a two-component ATP-dependent protease in which the ATPase HslU unfolds and translocates protein substrates and the HslV protease degrades the unfolded proteins [1]–[4]. HslVU was first identified in prokaryotes [1], [2], and has since been detected in many archaea and only recently in eukaryotes [5]–[12]. A catalytic threonine residue is located at the first N-terminal amino acid of HslV protease. HslV shares an approximately 20% sequence similarity and well-conserved folding pattern with the β-subunit of the eukaryotic 20S proteasome core particle [4], [13], [14]. HslU forms a hexameric ring and two hexameric HslUs cap the ends of a double-donut shaped dodecameric HslV [4], [15]–[17]. HslU is responsible for protein unfolding by ATP hydrolysis and feeds the substrates into the HslV chamber. The C-terminal tail of HslU interacts with the interface between protomers of HslV to generate the active HslVU complex [18]–[20]. Interestingly, the interaction between HslU and HslV results in a mutual cross activation, inferring that these two enzymes allosterically communicate with each other [20]–[23].

**Figure 1:**
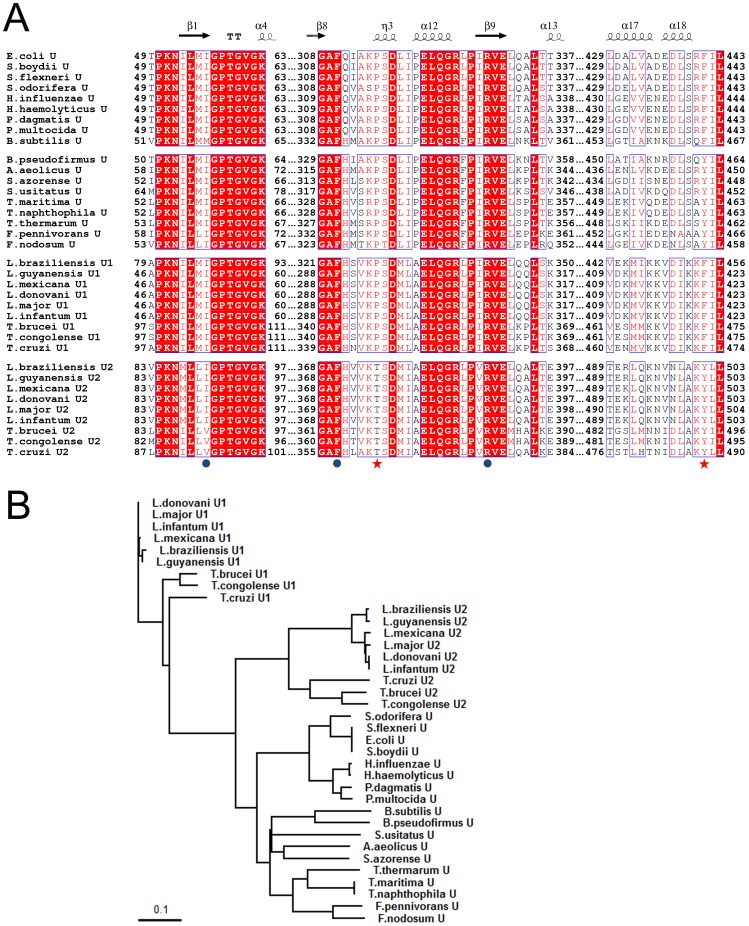
Sequence comparisons among HslU proteins from three different biological kingdoms. (A) Sequence alignment of C-terminal segment and its neighboring region in HslUs from different organisms (See Materials and Methods for detailed uniprot ID and species). The sequences are grouped in the order prokaryotic, archaeal, eukaryotic HslU1, and eukaryotic HslU2. Shading indicates residues that are identical (bold white in red-shaded box) or highly conserved (red in empty box) between species. Secondary structural elements are indicated above the sequence (α-helix, spring; β-strand, arrow). The mutated residues in this study are marked using a red star and the neighboring residues are marked using blue filled circles at the bottom. The sequence numbers for the aligned residues are also provided. (B) Phylogenetic tree of the aligned HslUs from panel (A). Prokaryotic HslUs are more similar to archael HslUs than to eukaryotic HslUs. There are two eukaryotic HslUs, of which HslU1 is relatively distant from the HslUs in the other kingdoms. The scale bar indicates amino acid substitutions per site.

Recently, we reported that only one HslU (HslU2) of the two HslUs present in *T. brucei* (TbU1 and TbU2) activates the HslV protease in this species (TbV) [24]. The key determinant for the function of this eukaryotic TbU2 is Tyr494 (Phe441 in *E. coli*) located at the C-terminal tail which activates TbV. In most prokaryotic systems, the equivalent residue for this Tyr494 is a strictly conserved phenylalanine and eukaryotic HslU1s also possess a conserved phenylalanine at this position (Fig. 1A). To dissect the role of this bulky aromatic residue at the C-terminal tail of the HslUs, we initially performed a peptidase assay using HslV from *E. coli* (EcV) with a synthetic peptide containing tyrosine at the position 441. Surprisingly, the peptidase activity of EcV was markedly enhanced and we further performed a biochemical characterization of an F441Y mutant EcU. There is a proline residue at position 315 near Phe441 in the free HslU structure. This proline locates at the end of a loop (residues 312–314) facing HslV, which seems to be important for HslV interaction [25]. In prokaryotes, this proline is strictly conserved, but in eukaryotic HslU2 this residue is a threonine (Fig. 1A). Hence, we generated a P315T mutant as well as a P315T/F441Y double mutant and characterized these products using peptidase, caseinolysis, SulA degradation, and ATPase assays. In addition to these biochemical assays, the primary sequences of HslUs from multiple organisms in three different kingdoms were analyzed (Fig. 1B). Our current study thereby demonstrates the importance of the C-terminal tail of HslU and provides new insights into the molecular characteristics of this enzyme including the critical role of the protruded loop near proline 315. Our current findings thus shed important new light on the molecular evolutionary pathway of HslU.

## Materials and Methods

### Sample preparation

The cloning, expression, and purification of EcV and EcU were performed as described previously [14], [15], [24], [25]. The expression vectors for the inactive HslV mutant, ΔT1_EcV and the EcU mutants P315T, F441Y, and P315T/F441Y were generated using QuikChange site-directed mutagenesis (Stratagene) with templates containing wild-type EcV and EcU genes cloned into the pET-22b(+) and pET-12a vector, respectively. The following primers were used in the mutagenesis reactions: ΔT1_EcV (5′-GAAGAAGATATACATATGACAATAGTAAGCGTACGCCGTAA-3′; 5′-TTACGGCGTACGCTTACTATTGTCATATGTATATCTCCTTC-3′), for P315T or P315T/F441Y (5′-CAGGTCAGACGTTTTCGCAATCTGGAACGCGCC-3′; 5′-GGCGCGTTCCAGATTGCGAAAACGTCTGACCTG-3′) and for F441Y (5′-CCGGATCCTTATAGGATATAACGGCTCAGATCTTCAT-3; 5′-ATGAAGATCTGAGCCGTTATATCCTATAAGGATCCGG-3′). Mutagenesis was confirmed by DNA sequencing. The expression and purification of ΔT1_EcV, P315T, F441Y, and P315T/F441Y was carried out as for wild-type EcV and EcU.

For the efficient expression of MBP-SulA (maltose-binding protein fused with SulA), we constructed an ΔEcVU strain (*E. coli* BL21(DE3) *ΔhslU::amp, ΔhslV:cm*) using Red/ET Recombination methods and the pRedET vector [26], [27] purchased from Gene Bridges, GmbH. The pMal-p2-SulA plasmid was transformed into the *E. coli* ΔEcVU cells for overproduction of MBP-SulA in cultures grown at 25°C. The purification of MBP-SulA has been described previously [24], [28], [29].

### Peptide synthesis

C-terminal octapeptides were synthesized by JPT Peptide Technologies GmbH and included wild-type (EDLSRFIL) and mutant forms (EDLSRYIL).

### Biochemical assays

Peptidase was assayed using the chromogenic peptide, carbobenzoxy-Gly-Gly-Leu-7-amido-4-methyl coumarin (Z-GGL-AMC; Bachem) as a substrate [30]. Wild-type EcU, the P315T, F441Y, P315T/F441Y mutants and two synthetic octapeptides were used for EcV activation. The activity assay was conducted at 37°C in a buffer consisting of 20 mM Tris-HCl pH 7.7, 300 mM NaCl, 1 mM EDTA, 6.25 mM MgCl_2_, and 7.5% (v/v) dimethylformamide. When EcU or its mutant enzymes and not a peptide were added as an activator for EcV, ATP was added in the reaction mixture. The release of 7-amido-4-methyl-coumarin (AMC), which is dependent on peptidase activity, was monitored as reported previously [24]. For the caseinolytic activity assay, resorufin-labeled casein purchased from Roche Diagnostics, GmbH was used as the substrate [31]. The procedure for activity measurements followed the instructions provided by the manufacturer. For protein substrate degradation, MPB-SulA was used as described previously [7], [24], [32], [33] with some modifications. The ATPase activity of EcU or its mutants was also assayed as described previously [25], [34]. Proteins were quantified by the method of Bradford using bovine serum albumin as a standard [35].

### Molecular modeling

A molecular model of EcV-activation of EcU was generated using the structure of HslU in the HslVU complex from *Haemophilus influenzae* as a template [18]. The alteration of this structure was then realigned with the sequence of EcU. The initial model was energy-minimized with SPBDV [36].

### Sequence analyses and phylogenetic tree

BLAST searches were carried out on the UniProt website (http://www.uniprot.org/). Sequence alignment and phylogenetic analyses were performed at standard settings using the Clustal Omega website (http://www.ebi.ac.uk/Tools/msa/clustalo/). Tree reconstruction was obtained using the Neighbor-joining method and the tree visualization results from TreeView (version 1.6.6; http://taxonomy.zoology.gla.ac.uk/rod/treeview.html). The protein sequences used were as follows: UniProt ID P0A6H5 (*Escherichia coli* HslU), E7SX86 (*Shigella boydii* HslU), P0A6H7 (*Shigella flexneri* HslU), D4DZ19 (*Serratia odorifera* HslU), P43773 (*Haemophilus influenza* HslU), I3DSM1 (*Haemophilus haemolyticus* HslU), C9PP45 (*Pasteurella dagmatis* HslU), H8IG73 (*Pasteurella multocida* HslU), E8VAJ1 (*Bacillus subtilis* HslU), D3FT45 (*Bacillus pseudofirmus* HslU), Q9WYZ2 (*Thermotoga maritime* HslU), D2C619 (*Thermotoga naphthophila* HslU), F7YXR0 (*Thermotoga thermarum* HslU), H9UA01 (*Fervidobacterium pennivorans* HslU), A7HK26 (*Fervidobacterium nodosum* HslU), O66574 (*Aquifex aeolicus* HslU), C1DWL5 (*Sulfurihydrogenibium azorense* HslU), Q01Q20 (*Solibacter usitatus* HslU), A4H7X9 (*Leishmania braziliensis* HslU1), A4H5J8 (*Leishmania braziliensis* HslU2), E9BC50 (*Leishmania donovani* HslU1), E9BS7 (*Leishmania donovani* HslU2), S0CVC5 (*Leishmania guyanensis* HslU1), S0CS82 (*Leishmania guyanensis* HslU2), A4HWA6 (*Leishmania infantum* HslU1), Q8I0E1 (*Leishmania infantum* HslU2), Q4QFH5 (*Leishmania major* HslU1), Q4QI03 (*Leishmania major* HslU2), E9AQ06 (*Leishmania Mexicana* HslU1), E9AMM2 (*Leishmania mexicana* HslU2), Q57VB1 (*Trypanasoma brucei* HslU1), Q382V8 (*Trypanosoma brucei* HslU2), G0UMN2 (*Trypanasoma congolense* HslU1), G0V2B6 (*Trypanasoma congolense* HslU2), Q4DEP1 (*Trypanasoma cruzi* HslU1), and Q4DRN5 (*Trypanasoma cruzi* HslU2).

## Results

### The peptidase activity of EcVU complex is augmented by an F441Y mutation in EcU

**Figure 2:**
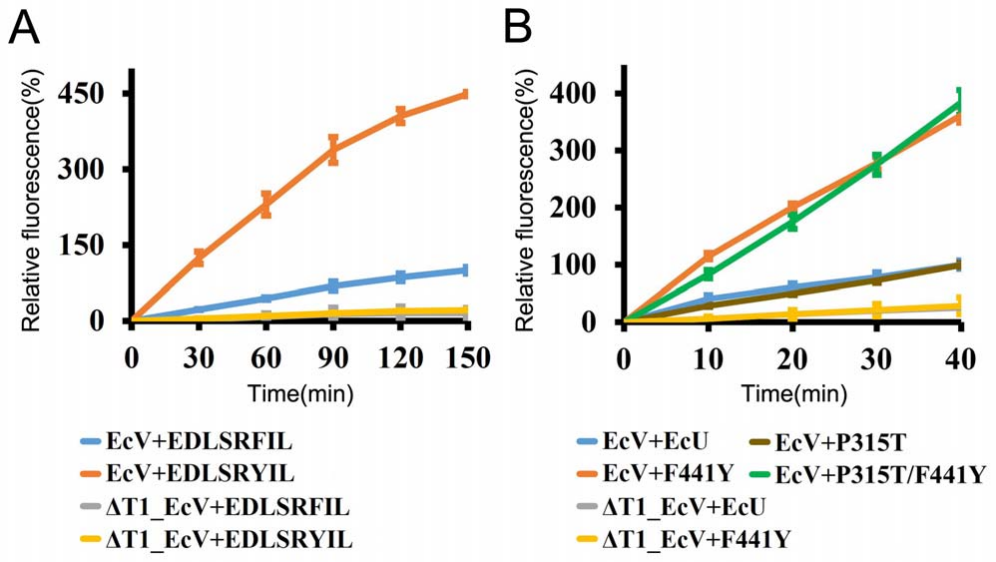
Peptidase activity assay. (A) The peptidase activity of EcV with octapeptide activators. The blue and orange lines represent the activity of EcV in presence of EDLSRFIL and EDLSRYIL peptides, respectively. The gray and yellow lines represent the activity of inactive ΔT1_EcV in presence of the same peptides. (B) Peptidase activity of the active form of EcV with EcU protein activators. The blue and orange lines represent the activity of EcV in the presence of wild-type EcU and F441Y mutant, respectively. The gray and yellow lines represent the activity of inactive ΔT1_EcV in presence of the same proteins. The brown and green lines represent the activity of EcV in the presence of P315T and P315T/F441Y double mutant, respectively. The error bars were calculated based on three independent experiments. The values are the means ± SD (n = 3).

The peptidase activity of HslV can be observed in the presence of the HslU activator and a synthesized octapeptide corresponding to the C-terminal eight amino acids of HslU can also activate the HslV peptidase activity [19], [20]. The C-terminal last eight residues of EcU are EDLSRFIL, and are well conserved in many other HslUs from prokaryotes such as *Haemophilus*, *Pasteurella*, *Serratia*, and *Shigella* (Fig. 1A). There are two HslU homologs in the mitochondria of *T. brucei*, an eukaryote, and the octapeptides corresponding to C-termini of TbU1 and TbU2 are VDIFFFIL and IDLAKYIL respectively [10]. Only the C-terminal peptide of TbU2 is involved in peptidase activation with TbV and the 3^rd^ tyrosine residue from the C-terminus is a key determinant of activation. This was confirmed by peptidase activity assay in which a phenylalanine was replaced by tyrosine in the amino acid sequence derived from the C-terminal octapeptides of EcU and TbU1 with TbV [24]. To further assess the importance of this tyrosine residue, we measured the peptidase activity of EcV in the presence of EDLSRFIL and EDLSRYIL peptides, as assayed using a synthetic substrate, Z-GGL-AMC. Surprisingly, the activity of EcHslV was elevated by more than three–fold by the mutated EDLSRYIL peptide (the underlined residue was originally phenylalanine; Fig. 2A). To further confirm the peptidase activity of EcVU, we mutated the phenylalanine residue of EcU to tyrosine (F441Y mutant). Similar to the peptide activator, the F441Y mutant strongly augmented the peptidase activity of EcV (Fig. 2B). To rule out any artificial activation of EcV, we generated the mutant ΔT1_EcV, in which we deleted the catalytic threonine residue at the N-terminus. As expected, this mutant enzyme possesses no peptidase activity (Fig. 2B).

### The caseinolytic activity of EcVU complex is also augmented by the EcU F441Y mutation

**Figure 3:**
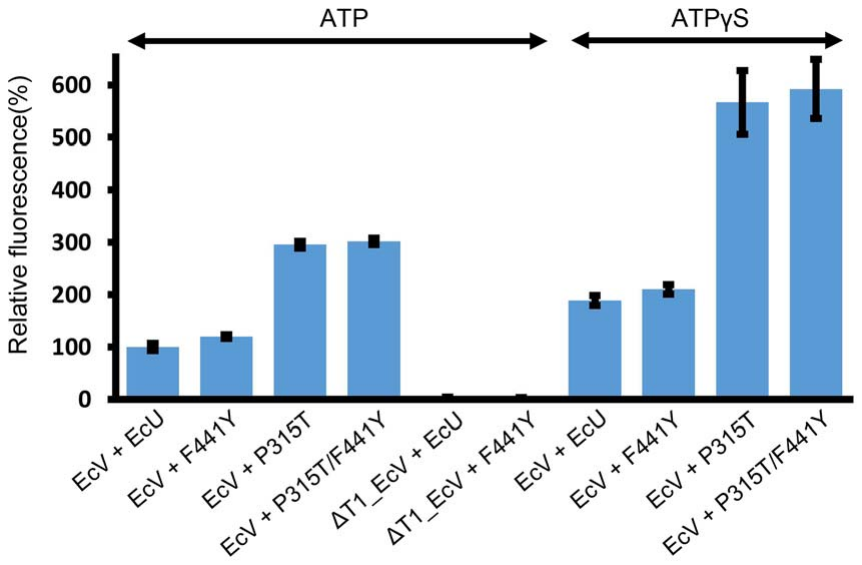
Caseinolytic activity of EcVU complex. Active wild-type EcV as well as inactive ΔT1_EcV were used in control experiments. Wild-type and three mutant EcU proteins (F441Y, P315T, and P315T/F441Y) were used to activate EcV in the presence of ATP or ATPγS as indicated above. Note that the caseinolytic activity of EcVU complex with ATPγS is higher than that with ATP, however, the general trend is basically the same with the three mutants. The caseinolytic activity of wild-type EcVU complex at a fixed time point (12 h) was arbitrarily set at 100%. Each bar and line represents the mean and standard deviation values from three independently performed assays.

The ATP-dependent proteases including HslVU complex behave differently for different substrates because peptide substrates do not require an unfolding step that uses ATP hydrolysis energy [25], [37], [38]. Casein, a model substrate for protease activity, is known to be only partially folded [39]. Hence, we measured the caseinolytic activity of the F441Y mutant in the presence of ATP or ATPγS. Casein degradation was further stimulated by the F441Y mutant compared with wild-type EcU (Fig. 3), but this was not a large increase (∼20% only) compared with peptide hydrolysis (Fig. 2A). There was a clear difference between casein and peptide substrates which freely pass through the entrance pore of HslV.

### The F441Y mutant has partially reduced ATPase activity

**Figure 4:**
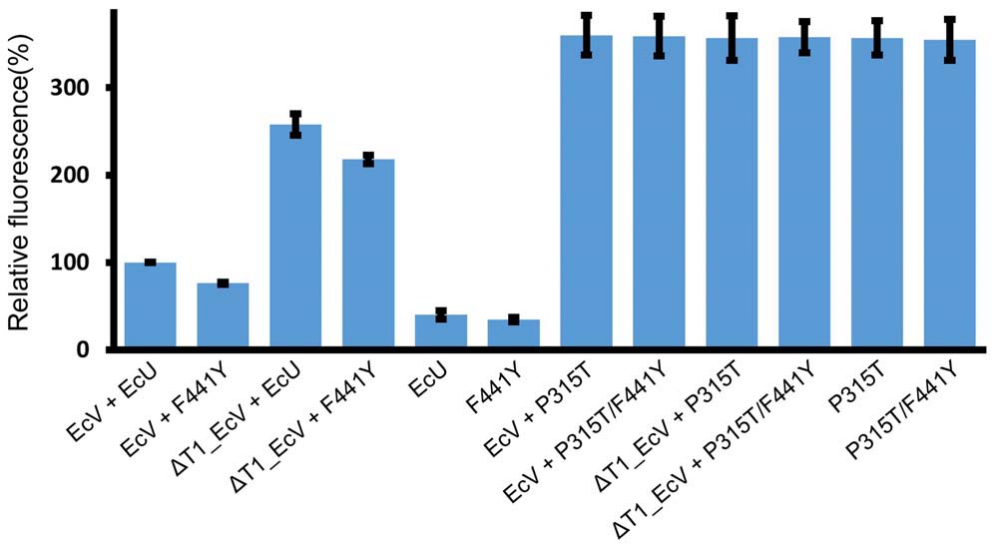
ATPase activity of EcU and its mutants in the presence or absence of EcV and ΔT1_EcV, respectively. The inactive ΔT1_EcV mutant was used in a control experiment as this variant is known to highly augment the ATPase activity of EcU [23]. Wild-type and three mutants (F441Y, P315T, and F441Y/P315T) were used for activating EcV. The ATPase activity of wild-type EcVU complex at a fixed time point (1 h) was arbitrarily set at 100%. Each bar and line represents the mean and standard deviation values from three independently performed assays.

ATPase and protease activities are tightly connected in two-component ATP-dependent proteases. As already reported, the HslVU complex shows approximately 3-fold higher ATPase activity than HslU alone [40]. Moreover, the inactive deletion mutant of the catalytic threonine residue of HslV is known to increase ATPase activity even further than active wild-type HslV [23]. Hence, we measured the ATPase activity of free EcU mutant and of EcU mutants in the presence of EcV or ΔT1_EcV mutant (Fig. 4). Unlike the enhancement of peptidase and caseinolytic activity by EcV-EcU F441Y mutant complex, the ATPase activity of F441Y decreased in the presence of either wild-type EcV or the inactive mutant form of ΔT1_EcV (Fig. 4). The free F441Y mutant also has marginally reduced ATPase activity (Fig. 4). Based on these results, it became clearer that the caseinolytic activities of EcVU or EcV-EcU F441Y complex are mostly independent of ATPase activity.

### The protein degradation activity of EcVU complex is not augmented by the F441Y mutation of EcU

**Figure 5:**
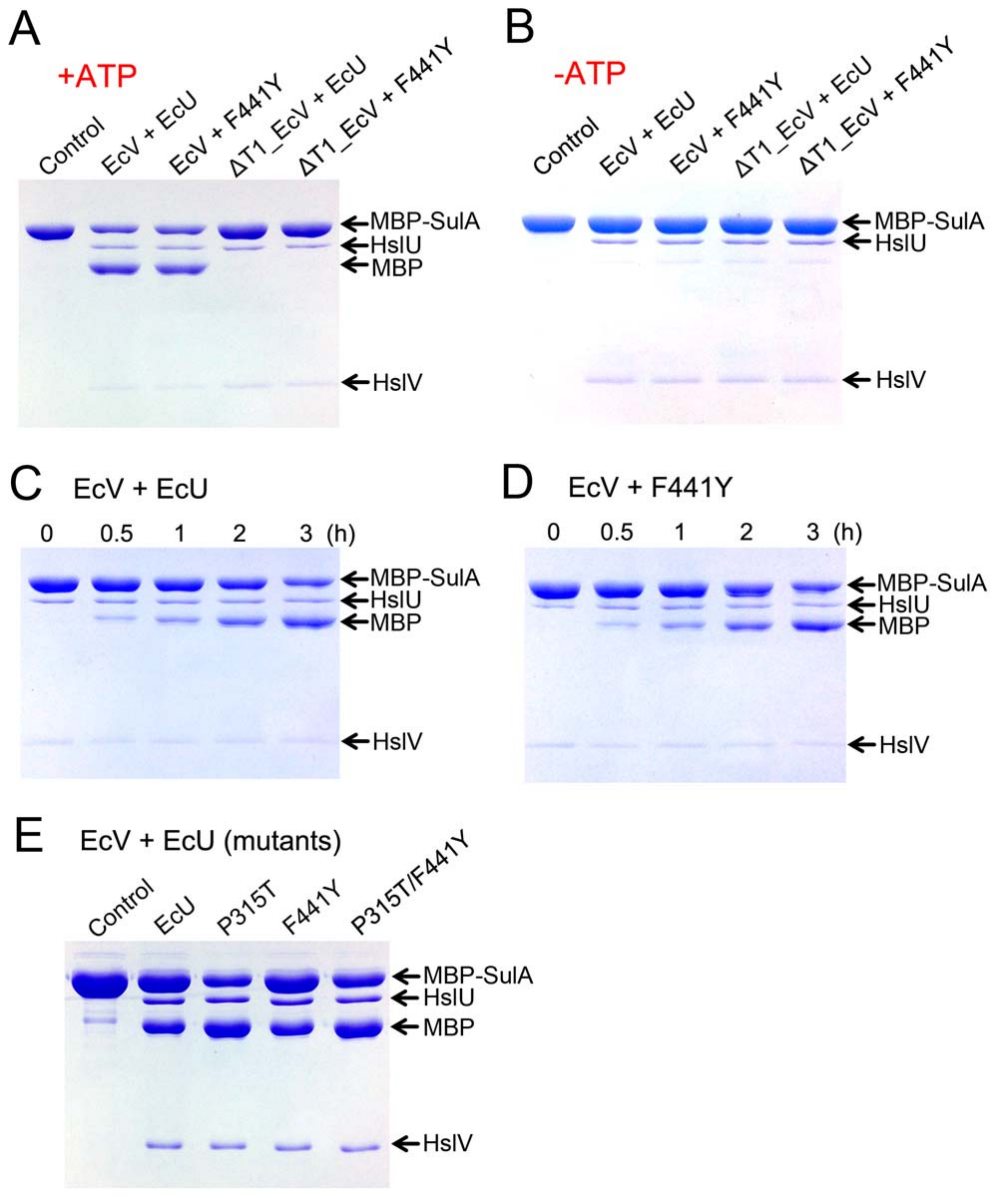
MBP-SulA degradation activity. (A) Bands for MBP-SulA (substrate), HslU (wild-type EcU or EcU mutants), MBP (reaction product), and HslV (EcV or ΔT1_EcV) are indicated. All reactions were performed in the presence of ATP and SulA is degraded in the reaction mixtures containing active EcV. The control lane contained substrate MBP-SulA only. (B) The same experiments were performed as described in panel (A) in the absence of ATP, and did not yield the MBP product. (C) Time course analysis of the degradation of MBP-SulA by EcV and EcU in the presence of ATP. (D) As in (C), except that the EcU F441Y mutant was used. The F441Y mutant produced no enhancement of SulA degradation compared with wild-type. (E) SulA degradation activity of wild-type EcVU and EcV-EcU mutant complexes at a fixed time point (2 h).

Next, we analyzed the degradation of a natural substrate SulA, which requires the ATPase activity of HslU [29]. The model protein substrate MBP-SulA is recognized by the I-domain of HslU, and is then unfolded and translocated into the catalytic pore of HslV by the ATPase, HslU [25]. Dissimilar to the results of our peptide hydrolysis and caseinolytic activity assays, we found no increase in protease activity when assayed using the F441Y mutant as an EcV activator (Fig. 5). The SulA degradation by EcVU complex clearly depended on the critical ATPase activity of EcU (Fig. 5B). For a better understanding of its different effects of the F441Y mutant on each type of activity, we performed structural analysis of the region close to the Phe441 residue of EcU.

### The HslV-activating C-terminal tail of HslU

**Figure 6:**
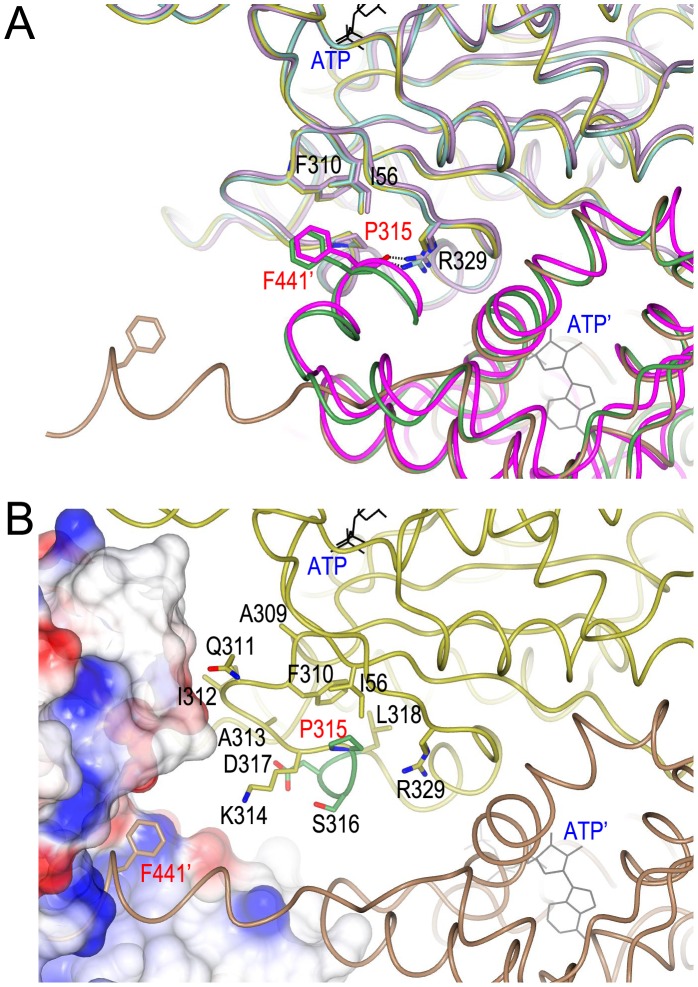
Structure of the EcU and EcV interaction region. (A) Schema showing the superimposed apo-subunit of EcU (PDB ID: 1DO0), ATP-bound subunit of EcU (PDB ID: 1DO0), and EcV-bound EcU, which reveals the extended C-terminal tail. This EcVU complex structural model was generated using the HiVU structure as a template [18]. In the apo EcU structure, one monomer of EcU is colored green, and its neighboring monomer is highlighted in sky blue for clarity. In the ATP-bound EcU structure, the two subunits are colored magenta and pink, respectively. In the schema for the HslVU complex, the two subunits are colored brown and yellow, respectively. The critical residues for the interaction between Phe441 and other amino acids are presented in a stick model. A prime annotation (′) is added for adjacent subunits of EcU. (B) The same view of the EcVU complex structure that includes the electrostatic potential of the EcV surface. The C-terminal tail of EcU extends toward a pocket formed by the two EcV subunits. The loop right before the Pro315 residue makes contact with EcV. The 3_10_-helix starting with Pro315 is colored differently. Oxygen and nitrogen atoms are colored red and blue, respectively.

Structurally, the C-terminal tail of HslU alone localizes at the cleft between HslU and a neighboring HslU and the Phe441 residue is close to several hydrophobic residues Ile56, Phe310 and Pro315 in the neighboring subunit, whilst the C-terminus of HslU in the active HslVU complex is involved in the interaction with HslV [14], [18]. Thus, the Phe441 residue in this C-terminal tail may be critical for structural changes in the enzyme and may also partially affects the ATPase activity of HslU, which is coupled with the complex formation [23]. We investigated the structure of the region near the Phe441 residue to examine why the F441Y mutation enhances peptide hydrolysis and casein degradation, but not ATPase hydrolysis and SulA degradation. Sequence analysis of the binding site of the EcV-activating C-terminal segment of EcU revealed several conserved residues (Fig. 1A) that are potentially critical for conformation changes during the HslU reaction cycle. This suggested that a transition occurs between a buried C-terminal segment within the free HslU molecule and the extended C-terminal tail for the HslV interaction (Fig. 6A). Among the residues analyzed, Pro315 seems to be very important because it shows a high degree of conservation that is comparable to the phenylalanine at the C-terminal tail in all prokaryotic HslUs (Fig. 1A). The eukaryotic HslU2 protein, a functional HslU that forms a complex with HslV in *T. brucei* [24], possesses a key tyrosine residue at the equivalent position to the phenylalanine in the C-terminal tail of EcU and has a conserved threonine residue at the equivalent position of the proline at position 315 (Fig. 1A). We thus generated an EcU P315T mutant.

### The P315T EcU mutant shows wild-type peptidase activity but marked enhancement of ATPase, caseinolytic, and SulA degradation activities

The peptidase activity of a P315T EcU mutant was assayed and as shown in Figure 2B, was found to be comparable to wild-type EcU. We measured the caseinolytic activity of this mutant and found a dramatic enhancement (Fig. 3). This markedly higher casein degradation activity of the P315T mutant is quite enigmatic because neither the peptidase nor caseinolytic functions of HslU require ATPase activity for the unfolding process. Thus, we evaluated the ATPase activity of this mutant and found it to be much higher than that of wild-type or F441Y proteins (Fig. 4). Accordingly, the degradation of the real substrate, SulA, was also found to be enhanced by EcV-P315T complex (Fig. 5E). This enhanced SulA degradation could have been readily explained by an augmented ATPase activity but the enhanced caseinolytic activity of this mutant would need to be reconciled. Although an unfolding process via ATP hydrolysis is not necessary, the ATP-binding of HslU triggers a conformational change in the C-terminal tail of HslU for HslV engagement. The loop region right before Pro315 is located between the ATP-binding site and C-terminal tail, and is also involved in the HslV interaction (Fig. 6A). Hence, the aforementioned loop region must cooperatively participate in HslV activation with the C-terminal tail. Subsequently, the catalytic threonine residue of HslV adopts a competent state through allosteric activation [21], [22].

### The P315T/F441Y double mutant shows an augmentation of peptidase, ATPase, caseinolytic, and SulA degradation activities

We generated a P315T/F441Y double mutant to determine whether it shows different enzymatic activity to each single mutant. The peptidase activity of this double mutant was similar to the F441Y single mutant, which is higher than wild-type (Fig. 2B). The other functional activities of HslU, i.e. caseinolytic, ATPase, and SulA degradation, were similar in the double mutant to those of the P315T mutant (Figs. 3–5). These results further indicated that the Pro315 and Phe441 residues have different roles in the regulation of HslU functional activity. Again, it was intriguing that Pro315 and Phe441 residues are proximal in the free HslU structure but distal in the HslVU complex (Fig. 6A) and play a critical role at different stages of the conformational changes in this complex coupled with its different enzymatic activities.

## Discussion

It is well known that the activities of HslVU complex are substrate dependent [1], [2], [25], [29], [41], [42]. In addition, except for ATP hydrolysis by HslU alone, the activities of this enzyme require communication between HslU and HslV. The hydrolysis of peptide substrates does not need ATPase activity [43], but requires ATP-binding to HslU to trigger a conformational change in its C-terminal tail to enable HslV interaction and subsequently to induce the allosteric activation of HslV. It must be noted in this regard that peptide substrates do not undergo a translocation step because they are sufficiently small to access the active site of HslV [44]. However, the disordered model substrate casein is different. Although, ATPase hydrolysis energy is not absolutely necessary for caseinolytic activity as it is for peptide hydrolysis (Fig. 3), casein still needs to pass through a narrow pore of the HslU hexamer and is therefore partly affected by the ATP binding of HslU. The degradation of the natural substrate SulA requires an unfolding step using ATP hydrolysis energy and these two processes are thus tightly coupled. Using this prior biochemical knowledge and previously reported structural data, we can explain the different characteristics of our HslU mutants at the molecular level.

The introduction of the hydroxyl group on the phenyl ring of Phe at residue 441 enhances the peptidase activity of HslVU complex dramatically but only marginally enhances its caseinolytic activity, and has no impact on its ATPase or SulA degradation activity (Figs. 2–5). One plausible explanation for this finding is that the F441Y mutation increases the binding affinity between HslU and HslV, and they then form a more stable active complex [24]. Peptide hydrolysis and caseinolytic activity enhancement would be expected if the active complex had a longer half-life. The structure of HslVU complex reveals that Phe441 in the C-terminal tail of HslU is distant from the ATP binding site (Fig. 6A). Hence, it would be expected to have no role in the ATP kinetics of the enzyme. However, the ATPase activity of HslVU is in fact partially reduced by the F441Y mutation. We thus speculate that this mutation partially disrupts the dynamic movement of the C-terminal tail from its buried conformation in free HslU to its conformation in the extended HslV-bound structure (Fig. 6A). This structural transition may well affect ATP hydrolysis also because the dynamic nature of the HslU molecule is significantly coupled with this enzymatic reaction.

We also analyzed the neighboring residue of Phe441 in term of its conformation in free HslU. The neighboring region is the loop between Ala309 and Lys314, and Pro315 is the first residue of a 3_10_-helix (Fig. 6B). This region is also relatively well conserved with a proline residue of interest at position 315 (Fig. 1A). In general, proline plays a critical role in the three dimensional structures of proteins because its side chain has a distinctive cyclic structure. It is also often present as the first residue of a helix, as is the case for Pro315 in HslU [45]. Sequence analysis of Pro315 showed that it is replaced by threonine in eukaryotic HslU2. The corresponding residues in prokaryotic and archaeal HslUs and eukaryotic HslU1 are conserved as proline (Fig. 1A). It was of interest that the P315T mutation enhances ATPase, caseinolytic, and SulA degradation activities of HslVU except for peptide hydrolysis, which is the opposite result to that of the F441Y mutant HslU. In addition, the double P315T/F441Y mutant showed highly enhanced peptidase, ATPase, caseinolytic, and SulA degradation activities, which are all of the functional assays we performed. Hence, it was necessary to elucidate why the P315T mutant behaves in this way. As shown in Figure 6B, the loop near to Pro315 is involved in HslV binding and previously, it has been reported that a penta-glycine insertion between the Gln311 and Ile312 residues in EcU resulted in an 80% activity reduction in a peptide degradation assay and 60∼80% loss of ATPase activity compared with wild-type EcU and EcVU complex [25]. Our current point mutation results and previous insertion mutant data clearly suggest that this region is critical for modulating the protease and ATPase activities of HslVU complex.

There is an analogy here with the ClpXP system [3], [37], [38], [46], as ClpX ATPase has two distinct binding regions with ClpP. It has been shown that the IGF loop of ClpX interacts statically with the peripheral hydrophobic regions of ClpP [47]. The IGF loops constitute the most protruding flexible parts of the ClpX hexamer and similarly, the C-terminal tail of HslU is located in the peripheral part of the hexameric HslU and fits into the primary pocket of HslV. Previously, the second region of ClpX involved in ClpP binding, the ‘pore-2’ loop (^191^RKSDNPSITRD^201^), has been reported to possess a dynamic nature [46]. Although the binding affinity of this region is not strong per se, it varies dynamically with the nucleotide state of individual HslU subunits, controls ATP-hydrolysis rates, and translocates the substrate efficiently. Hence, it is tempting to speculate that the C-terminal tail of HslU is a primary contact region with HslV that principally governs the binding affinity and correct orientation of the complex. However, the loop at the position just before the Pro315 residue plays a critical role in the additional interactions with HslV as well as in modulating the enzymatic activities of the resulting complex.

The ATP-dependent two-component HslVU proteases exist in all three biological kingdoms. The eukaryotic HslUs are classified into two groups, HslU1 and HslU2. Interestingly, the C-terminal tails of the HslU1s possess a phenylalanine at the equivalent position to prokaryotic HslUs. The importance of the tyrosine residue at this position in HslU2 from the mitochondria of an eukaryote, *Trypanosoma brucei* is known and this HslU2 forms a functional complex with HslV [24]. The residues nearby might therefore have coevolved for functional reasons. The Pro315 near to Phe441 in prokaryotic HslU proteins has been replaced with a threonine in eukaryotic HslU2, but is conserved in eukaryotic HslU1 (Fig. 1A). Hence, the proline residue at this position can be an additional indicator along with the tyrosine residue in the C-terminal tail for the selection of functional HslU molecules in the eukaryotic HslVU system. The archaeal HslUs fall between this arrangement because they harbor a proline residue at the equivalent position, but a tyrosine residue at the C-terminal tail. In the evolutionary pathway, the phenylalanine residue has probably been replaced with a tyrosine for tighter binding with HslV, whereas the proline residue in the activity modulating loop is conserved. It is interesting to note that archaeal HslU has no nucleotide specificity and is also active at high temperature [7]. This phenomenon is also found in the archaeal proteasome, in which the 20S proteasome core particle is activated by proteasome-activating nucleosidase [48]. We speculate that since the archaeal HslUs are active at high temperatures which allow enhanced dynamic movement of all atoms including the loop near Pro315, the replacement of threonine is not necessary for this activity. However, for eukaryotic HslU2, a more dynamic loop would possibly be necessary. Furthermore, when we analyzed the whole sequences of the HslUs, the archaeal HslUs were found to be closer to the prokaryotic HslUs than the eukaryotic HslU2s (Fig. 1B). However, eukaryotic HslU1 proteins are relatively distinct from the functional HslUs that forming HslVU complexes in prokaryotes, archaea, and eukaryotes. This comparative sequence analysis combined with our mutational analysis of EcU confirm that the Phe441 residue at the C-terminal tail and the Pro315 near the dynamic loop of HslU communicate to enable HslV activation and that they have evolved together to ensure the optimal activity of prokaryotic, archaeal, and eukaryotic ATP-dependent HslVU complexes.
